# Awareness and Knowledge of Women About Hormone Replacement Therapy in Taif City, Saudi Arabia

**DOI:** 10.7759/cureus.34423

**Published:** 2023-01-31

**Authors:** Hussam A Albaqami, Abdulelah A Alzahrani, Muhanna J Altalhi, Abdulrahman F Alsubaie, Hamad S Alshoail, Shatha H Alziyadi

**Affiliations:** 1 College of Medicine, Taif University, Taif, SAU; 2 Obstetrics and Gynecology, Taif University, Taif, SAU

**Keywords:** prevalence, taif city, breast cancer, saudi arabia, hormone replacement therapy

## Abstract

Background

It is crucial for women to have a good understanding of menopause from a young age, as this natural transition can have significant effects on their lives. Having this knowledge can help them handle the associated changes and improve their overall well-being. This study aimed to assess the level of awareness, attitude, and misconceptions regarding hormone replacement therapy (HRT) and menopause among women residing in the Taif region.

Methodology

This was a cross-sectional study conducted on the general population in Taif, Saudi Arabia, using an online self-administered questionnaire through Google Forms (Google Inc., Mountain View, CA, USA) from July 2022 to December 2022. The study included women aged between 40 and 65 years. A previously validated questionnaire was used for data collection, which assessed participants’ awareness and knowledge of hormone replacement therapy in Taif. A 2-point system was used to grade each variable, where 2 points were given for a correct answer, 0 for an incorrect answer, and 1 for a neutral answer. Consistent with prior use of the questionnaire, participants who correctly answered 75% were considered to have good knowledge and understanding of HRT. Statistical analysis was performed using the Statistical Package for the Social Sciences (SPSS) (IBM SPSS Statistics, Armonk, NY, USA).

Results

A total of 383 participants were enrolled in this study. The mean age of the participants was 48 ± 6.2 years (ranging from 40 to 65 years). The mean knowledge score about hormone therapy during menopause was found to be 1.9 ± 2.4 (ranging from 0 to 9) out of 10. Of these participants, 63 (16.4%) were considered to have good knowledge, while 320 (83.6%) had poor knowledge. Additionally, 95 (24.8%) participants agreed to hormone replacement therapy during menopause, 136 (35.5%) believed that the advantages outweigh the disadvantages, 74 (19.3%) believed that it decreased the risk of cardiovascular diseases, and 113 (29.5%) believed that it decreased the risk of osteoporosis. The study also found that employment status, previous knowledge about hormone replacement therapy, and current use of it were significantly associated with awareness about hormone replacement therapy (p-value = 0.025, <0.001, and 0.003, respectively), with employed participants, those who heard about it, and those who currently use it tending to have higher awareness level compared to others.

Conclusion

Our study found that there is a poor level of knowledge and awareness about menopause and hormone therapy among the participants. Employment status was found to be associated with the level of knowledge.

## Introduction

Menopause is a natural biological process in which all women transition from a reproductive to a non-reproductive state as a result of ovarian failure. The World Health Organization defines menopause as the permanent cessation of menstrual periods for 12 months or more due to the cessation of ovarian hormone production [1]. The age at which menopause begins can vary, but the mean age in the Kingdom of Saudi Arabia is 48.3 ± 3 years, as per research [2].

The use of hormone replacement therapy (HRT) has undergone significant changes since its introduction in the 1940s. The initial findings of the Women’s Health Initiative, published in 2004, showed an increased risk of coronary heart disease (CHD) and breast cancer, which led to a dramatic decrease in the use of HRT [3]. However, recent reanalysis of data with age stratification, newer randomized and observational studies, and several meta-analyses have consistently shown reductions in CHD and overall mortality when HRT is initiated soon after menopause. Therefore, the trend in the acceptance of HRT use after menopause has been swinging back [4].

Hormone replacement therapy (HRT) is known to help relieve several menopausal symptoms such as hot flashes, night sweats, mood swings, vaginal dryness, and reduced sex drive, as well as prevent postmenopausal osteoporosis. However, a study published in the Saudi Medical Journal found that awareness of HRT among the general female population was low, at 26.7%, and the use of HRT was even lower, at 5% [5].

With the low levels of awareness and usage of HRT among the general female population, this proposed study aims to evaluate HRT awareness among women in the Taif region. The study aims to compare the results with the previously conducted study to better comprehend the degree of HRT awareness and also determine what steps need to be taken to enhance HRT knowledge.

## Materials and methods

This study is a descriptive cross-sectional study conducted in Taif, Saudi Arabia, from July 2022 to December 2022. The study included women aged between 40 and 65 years old from Taif, Saudi Arabia, and excluded women from outside Taif, those under 40 years old, and those over 65 years old.

The sample size was calculated using the Epi Info program (Centers for Disease Control and Prevention, Atlanta, GA, USA), based on a 95% confidence interval, a 5% margin of error, and the total selected population of women in Taif, Saudi Arabia. The estimated sample size was 380, and it was adjusted to 420 to compensate for a 10% non-response rate.

The study was conducted using an online self-administered questionnaire via Google Forms (Google Inc., Mountain View, CA, USA). The generated link was randomly shared on various social media platforms (Facebook, WhatsApp, Telegram, and Twitter). The aim of the study was clearly explained in the interface, and a convenient non-probability sampling technique was employed to collect data from the participants. A validated questionnaire was used, based on Bakarman’s study questionnaire [5,6]. The questionnaire included sociodemographic characteristics of the participants such as age group, sex, nationality, education status, body mass index (BMI), and residence. It also included questions regarding the awareness and knowledge of women about hormone replacement therapy in Taif City. A common grading method was used for each variable in the questionnaire: 2 points for the correct option, 0 for the incorrect answer, and 1 for a neutral answer. A participant who correctly answered 75% or more of the questions (27 out of 36) was considered to have good knowledge and practice about HRT.

The study received respective approval from the Research Ethics Committee of Taif University with reference number HAO-02-T-105. All data will be kept confidential and used only for research purposes. The questionnaire was pretested in a pilot study with a sample of 20 participants, whose results were included in the final study. Some modifications were made accordingly to ensure clarity and easy understanding of the questions.

The collected data were processed using the Statistical Package for the Social Sciences (SPSS) version 26 (IBM SPSS Statistics, Armonk, NY, USA) software. Percentages were given for qualitative variables, and the mean ± standard deviation (SD) was given for quantitative variables. The independent sample t-test (for two groups) was used to compare numerical variables and determine significance. The chi-square test was used to compare categorical variables. A p-value of ≤0.05 was considered statistically significant in all statistical analyses.

## Results

A total of 420 questionnaires were distributed, and 383 participants completed the study, resulting in a response rate of 91.19%. The mean age of the participants was found to be 48 ± 6.2 years (ranging from 40 to 65 years), and their average body mass index (BMI) was 28.8 ± 6.1 kg/m^2^. In terms of educational level, 243 (63.4%) had a university education or higher, 80 (20.9%) had a high school education, 35 (9.1%) had an intermediate school education, 19 (5%) had an elementary school education, and six (1.6%) had no formal education. Additionally, 176 (46%) were employed, 166 (43.3%) were unemployed, and 41 (10.7%) were retired. The majority, 317 (82.8%), were married, 27 (7%) were divorced, 22 (5.7%) were widowed, and 17 (4.4%) were single (Table 1).

**Table 1:** Sociodemographic characteristics of the respondents (n = 383) SD: standard deviation, BMI: body mass index

Variable	Mean ± SD	Range
Age (years)	48 ± 6.2	40-65
Height (cm)	159 ± 7.8	110-195
Weight (kg)	72.5 ± 13.8	40-140
BMI (kg/m^2^)	28.8 ± 6.1	16.5-69.4
	Frequency	Percent
Educational level		
No formal education	6	1.6%
Elementary school	19	5%
Intermediate school	35	9.1%
High school	80	20.9%
University or higher	243	63.4%
Employment status		
Employed	176	46%
Not employed	166	43.3%
Retired	41	10.7%
Marital status		
Single	17	4.4%
Married	317	82.8%
Divorced	27	7%
Widowed	22	5.7%

The majority, 194 (50.7%), were premenopausal, and 193 (50.4%) had previously used contraceptives in the past. About 22 (5.7%) participants were current smokers. Additionally, 106 (27.7%) had previously heard about menopausal hormone therapy, while 277 (72.3%) had not. Seven (1.8%) participants were currently using menopausal hormone therapy, 17 (4.4%) had used it in the past, and 359 (93.7%) had never used it. Of those who were not currently using menopausal hormone therapy, 102 (27.9%) were considering using it in the future. Overall, 115 (30%) would recommend using menopausal hormone therapy (Table 2).

**Table 2:** History of contraceptives and menopausal hormone therapy

Variable	Category	Frequency	Percent
Menopausal status	Premenopausal	194	50.7%
	Perimenopausal	108	28.2%
	Postmenopausal	72	18.8%
	Surgical menopause	9	2.3%
Have you been using any type of contraceptive?	Currently using it	57	14.9%
	Used it in the past	193	50.4%
	Never used it	133	34.7%
Are you a smoker?	Yes	22	5.7%
	No	361	94.3%
Have you ever heard about menopausal hormone therapy?	Yes	106	27.7%
	No	277	72.3%
Have you been using menopausal hormone therapy?	Currently using it	7	1.8%
	Used it in the past	17	4.4%
	Never used it	359	93.7%
If menopausal hormone therapy is used, please select the duration (n = 24).	1-6 months	6	25%
	6-12 months	4	16.7%
	1-2 years	5	20.8%
	More than two years	9	37.5%
If you did not use menopausal hormone therapy in the past or currently using menopausal hormone therapy, do you consider using menopausal hormone therapy in the future (n = 366)?	Yes	102	27.9%
	No	235	64.2%
	I don’t know	29	7.9%
Do you recommend using menopausal hormone therapy?	Yes	115	30%
	No	234	61.1%
	I don’t know	34	8.9%

About 126 (50.4%) participants were found to be using combined pills, 80 (32%) were using copper and intrauterine devices, 45 (18%) were using hormonal intrauterine devices, 34 (13.6%) were using mini-pills (progesterone-only pills), 22 (8.8%) were using contraceptive patches, 14 (5.6%) were using contraceptive injections, and two (0.8%) were found to be using contraceptive implants. The source of information regarding menopausal hormone therapy was the internet for 101 (59.1%) of 171 participants, a doctor or healthcare provider for 47 (27.5%), a friend or colleague for 43 (25.1%), and the media for 31 (18.1%). However, 212 (55.4%) participants had never heard of menopausal hormone therapy.

The mean knowledge score about menopausal hormone therapy was found to be 1.9 ± 2.4 (range: 0-9) out of 10. Approximately 63 (16.4%) participants were considered to have good knowledge, while 320 (83.6%) participants were considered to have poor knowledge about menopausal hormone therapy. Approximately 95 (24.8%) agreed on hormone replacement during menopause. Seventy-four (19.35%) agreed that menopausal hormone therapy can decrease the risk of cardiovascular disease. A total of 113 (29.5%) participants agreed that it decreases the risk of developing osteoporosis. Additionally, 291 (76%) did not know that menopausal hormone therapy decreases flushes and night sweats. Furthermore, 298 (77.8%) did not know that it can decrease the risk of colon cancer. Overall, 224 (58.5%) participants did not know if menopausal hormone therapy is good for menopausal symptoms (Table 3).

**Table 3:** MHT knowledge of the study participants MHT: menopausal hormone therapy

Statement	Agree (number (%))	Disagree (number (%))	I don’t know (number (%))
It can replace decreased hormones during menopause.	95 (24.8)	31 (8.1)	257 (67.1)
It can decrease the risk of cardiovascular disease.	74 (19.3)	37 (9.7)	272 (71)
It can decrease the risk of developing osteoporosis.	113 (29.5)	11 (2.9)	259 (67.6)
It can decrease hot flashes and night sweats.	74 (19.3)	18 (4.7)	291 (76)
It can decrease the risk of colon cancer.	53 (13.8)	32 (8.4)	298 (77.8)
Menopausal hormone therapy is a good option if you have menopausal symptoms.	113 (29.5)	46 (12)	224 (58.5)
Menopausal hormone therapy is good for some women.	136 (35.5)	18 (4.7)	229 (59.8)
The advantages of menopausal hormone therapy exceed the disadvantages.	69 (18)	38 (9.9)	276 (72.1)
It can increase the risk of cardiovascular disease.	41 (10.7)	44 (11.5)	298 (77.8)
It can increase the risk of breast cancer.	50 (13.1)	45 (11.7)	288 (75.2)
It can increase the risk of cervical cancer.	50 (13.1)	41 (10.7)	292 (76.2)
It can cause vaginal bleeding.	45 (11.7)	43 (11.2)	295 (77)
It has many complications and side effects.	73 (19.1)	35 (9.1)	275 (71.8)
The disadvantages of menopausal hormone therapy exceed the advantages.	44 (11.5)	47 (12.3)	292 (76.2)
Menopausal hormone therapy should be avoided.	70 (18.3)	58 (15.1)	255 (66.6)

Employment status was found to be significantly associated with awareness about hormone replacement therapy (p-value = 0.025), with employed participants tending to have higher levels of knowledge and awareness than others. Hearing about menopausal hormone therapy and level of awareness about menopausal hormone therapy were found to be significantly associated (p-value < 0.001), with those who had heard about it tending to have higher levels of awareness compared to others. Using menopausal hormone therapy was found to be significantly associated with awareness about menopausal hormone therapy (p-value = 0.003), with those currently using menopausal hormones tending to have higher levels of awareness than others. Age, BMI, educational level, marital status, menopausal status, and contraceptive usage were not found to be significantly associated with the awareness and knowledge of women about hormone replacement therapy (p-values = 0.053, 0.327, 0.153, 0.265, 0.635, and 0.596, respectively) (Table 4).

**Table 4:** Factors associated with awareness and knowledge of women about hormone replacement therapy *Statistically significant (p < 0.05) SD: standard deviation, BMI: body mass index

Variable		Awareness		p-value
		Good (mean ± SD)	Poor (mean ± SD)	
Age (years)		46.6 ± 6	48.2 ± 6.2	0.053
BMI (kg/m^2^)		28.1 ± 4.8	28.9 ± 6.3	0.327
		Good (number (%))	Poor (number (%))	p-value
Educational level	Illiterate	0 (0)	6 (100)	0.153
	Elementary school	3 (15.8)	16 (84.2)	
	Intermediate school	1 (2.9)	34 (97.1)	
	High school	15 (18.8)	65 (81.3)	
	University or higher	44 (18.1)	199 (81.9)	
Employment status	Employed	35 (19.9)	141 (80.1)	0.025*
	Not employed	27 (16.3)	139 (83.7)	
	Retired	1 (2.4)	40 (97.6)	
Marital status	Single	5 (29.4)	12 (70.6)	0.265
	Married	47 (14.8)	270 (85.2)	
	Divorced	6 (22.2)	21 (77.8)	
	Widowed	5 (22.7)	17 (77.3)	
Menopausal status	Premenopausal	32 (16.5)	162 (83.5)	0.635
	Perimenopausal	21 (19.4)	87 (80.6)	
	Postmenopausal	9 (12.5)	63 (87.5)	
	Surgical menopause	1 (11.1)	8 (88.9)	
Contraceptive usage	Currently using it	12 (21.1)	45 (78.9)	0.596
	Used it in the past	30 (15.5)	163 (84.5)	
	Never used it	21 (15.8)	112 (84.2)	
Have you ever heard about menopausal hormone therapy?	Yes	31 (29.2)	75 (70.8)	<0.001
	No	32 (11.6)	245 (88.4)	
Have you been using menopausal hormone therapy?	Currently using it	4 (57.1)	3 (42.9)	0.003*
	Used it in the past	6 (35.3)	11 (64.7)	
	Never used it	53 (14.8)	306 (85.2)	
If menopausal hormone therapy is used, please select the duration (n = 24).	1-6 months	2 (33.3)	4 (66.7)	0.002*
	6-12 months	2 (50)	2 (50)	
	1-2 years	4 (80)	1 (20)	
	More than two years	2 (25)	6 (75)	

## Discussion

Assessing the level of knowledge and awareness regarding menopausal hormone replacement is important, as it can affect women’s decisions to start hormone replacement therapy [7,8]. The current study examined the awareness, mentality, and misconceptions about hormone replacement therapy and menopause in women who live in the Taif region.

Only less than one-third (27.7%) of the participants had previously heard about menopausal hormone therapy, which is much lower than the 67% reported in a parallel study conducted by Lydakis et al. [9]. Only 1.8% of the participants were currently using menopausal hormone therapy, 4.4% had used it in the past, and the rest had never used it. Similar findings were reported in a parallel study conducted by Nagata et al., in which 2.5% of women reported current use of menopausal hormone therapy and 6.3% had previously used it [10]. The most commonly reported duration of using menopausal hormone therapy was found to be more than two years, consistent with the findings of a congruent study conducted in the UK, in which the mean duration of menopausal hormone therapy was found to be 1.4 years [11].

Out of a total of 10, the mean knowledge score about menopausal hormone therapy was found to be 1.9. Only 16.4% were considered to have good knowledge, while the vast majority (83.6%) had poor knowledge about menopausal hormone therapy. These findings were in contrast to those of a parallel study conducted by Maharaj et al., in which more than half of the participants had a good awareness of menopausal hormone therapy [12].

Less than one-fifth (19.3%) agreed that menopausal hormone therapy can decrease the risk of cardiovascular disease. These findings were similar to those of a study conducted in the USA, which demonstrated a favorable effect of menopausal hormone replacement therapy, but it should not be considered as primary or secondary prevention for cardiovascular diseases [13]. About less than one-third (29.5%) of the participants agreed that it decreases the risk of developing osteoporosis, which is a lower percentage compared to a parallel study carried out by Alharthi et al., in which 66.3% of the participants were aware that menopausal hormone therapy decreases the risk of osteoporosis [14].

More than one-third (35.5%) of the participants agreed that the advantages of menopausal hormone therapy outweigh the disadvantages. These findings were consistent with those of the study by Blümel et al., in which most participants using menopausal hormone replacement therapy had a positive perception of their health and would not recommend avoiding menopausal hormone replacement therapy [15].

Employment status was found to be significantly associated with awareness about hormone replacement therapy, with employed participants tending to have higher levels of knowledge and awareness than others. Using menopausal hormone therapy was also found to be significantly associated with awareness about menopausal hormone therapy, with those currently using menopausal hormones tending to have higher levels of awareness than others. These findings were supported by the study by Hamid et al., in which educational level was found to be significantly associated with awareness about menopausal hormone replacement therapy [16].

The study has certain limitations as this was a cross-sectional study, which only evaluated the situation at one point in time and not across a period of time, and may not have been able to capture the changes in knowledge and awareness over time. The other limitation was selection bias, as the sample may not have been representative of all women in Taif City, due to the method of recruitment or self-selection of participants.

## Conclusions

Our study revealed that there is a lack of knowledge about menopause and menopausal hormone therapy among the participants. This is a concern as knowledge about these topics can influence women’s decisions to start hormone replacement therapy. Developing and promoting health education campaigns that provide accurate and reliable information about menopause and hormone replacement therapy can help increase awareness and understanding among women.
